# Supplementary material for: Donated Blood Screening for HIV, HCV and HBV by ID-NAT and the Residual Risk of Iatrogenic Transmission in a Tertiary Care Hospital Blood Bank in Puebla, Mexico
Source: Viruses. 2023 Jun 6;15(6):1331. doi: 10.3390/v15061331 (PMC10305412; doi:10.3390/v15061331)
Supplement: Supplementary file 1 [file viruses-15-01331-s001.zip › Table S1.pdf]

Table S1. Blood donors with more than one marker during the study periods

2012-2015

**Serological tests**				**NAT**			**Number of DB**
Anti-HIV/p24	*Anti-HCV*	*HBsAg*	*Anti-HBc*	*HIV*	*HCV*	*HBV*	-
R	R	NR	R	P	N	P	1
R	NR	NR	R	P	N	N	3
NR	R	R	NR	N	P	N	1
NR	R	NR	R	N	P	N	1
Total							6

2017-2019

**Serological tests**				**NAT**			**Number of DB**
Anti-HIV/p24	*Anti-HCV*	*HBsAg*	*Anti-HBc*	*HIV*	*HCV*	*HBV*	-
R	NR	NR	R	P	N	N	2
R	NR	NR	R	N	N	P	1
R	NR	NR	R	P	N	N	4
NR	R	NR	R	N	N	N	3
R	R	NR	R	P	N	N	2
NR	NR	R	R	N	P	P	1
NR	NR	R	R	N	P	N	7
Total							20

R – reactive; NR – nonreactive; P – positive; N- negative.
