# Supplementary material for: Donated Blood Screening for HIV, HCV and HBV by ID-NAT and the Residual Risk of Iatrogenic Transmission in a Tertiary Care Hospital Blood Bank in Puebla, Mexico
Source: Viruses. 2023 Jun 6;15(6):1331. doi: 10.3390/v15061331 (PMC10305412; doi:10.3390/v15061331)
Supplement: Supplementary file 1 [file viruses-15-01331-s001.zip › Table S2.pdf]

Table S2. Seroprevalence in blood donors reported by other studies carried out in México during the period covered by the study 2012-2015 and 2017- 2019.

Reference	Rojo-Medina et al.	Robles-Martínez et al.	Hernández-Romano et al.	Navarrete-Castro et al.	Portillo-García et al.	Aguirre-Orozco et al.	González-Santos et al.	Guerrero-García et al.	Prevalence mean (CI 95%)
Year of publication	2017	2016	2016	2017	2018	2019	2019	2021	
Study period	2012	2014	2014-2015	2012-2015	2017-2018	2015-2018	2016-2019	2018-2019	
Blood bank	CETS, IMSS, private	CETS	CETS	ISSSTE	CETS	CETS	IMSS	IMSS	
Federal entity	National	Jalisco	Veracruz	CdMx	Chihuahua	Jalisco	Nuevo León	Jalisco	
Number of BD	1,782,890	133,393	9,817	36,793	41,387	36,065	188,705	80,391	
Prevalence serological markers									
Combo anti-HIV/ p24Ag N (P)	TNIS	133 (0.10)	TNIS	67 (0.18)	56 (0.13)	350 (0.10)	214 (0.11)	152 (0.19)	0.13 (0.092-0.16)
Anti-HCV N (P)	10,217 (0.57)	760 (0.57)	TNIS	180 (0.49)	TNIS	153 (0.43)	918 (0.48)	385 (0.48)	0.50 (0.44-0.56)
HBsAg N (P)	2,675 (0.15)	160 (0.12)	21 (0.22)	42 (0.11)	TNIS	57 (0.15)	256 (0.13)	181 (0.22)	0.15 (0.11-0.20)
Anti-HBc N (P)	TNIS	TNIS	1.13 (1.15) - 1.17	TNIS	TNIS	TNIS	TNIS	TNIS	

Prevalence of serological test in BD reported by other studies carried out in México during the period covered by the study 2012-2019. CETS = State Blood Transfusion Centers (Jalisco); IMSS= Mexican Social Security Institute (Jalisco and Nuevo León); ISSSTE= Institute of Social Security and Services of State Workers (Mexico City); TNIS=test not included in the study; N (P) = number (prevalence).

References

Rojo-Medina, J.; Bello-López, J. M., National prevalence of hepatitis C and B viruses in Mexican blood donors, 2000–2012. Revista Médica del Hospital General de México 2017, 80, (1), 37-44.

Robles Martínez, A. K.; Becerra Leyva, M. G.; Licón González, G. E., Seroprevalencia de marcadores infecciosos en los servicios de Medicina Transfusional Públicos e Institucionales del Estado de Jalisco durante 2014. Rev Mex Med Transfus 2016, 8, (Supl. 1), S15.

Hernández-Romano, P. A.; Bravo-Sarmiento, E.; López-Balderas, N. A., Hepatitis B oculta en donadores del CETS-Veracruz. Rev Mex Med Tran 2016, 9, (1-2), 9.

Navarrete-Castro, J.; Siria Torreblanca, N.; Lebrija Córdova, V.; Carmona García, R.; De la Fuente Dorado, L.; González Avante, M., Prevalencia de agentes infecciosos transmisibles en donadores de hemocomponentes del Banco de Sangre del CMN «20 de Noviembre», ISSSTE Juan Navarrete Castro. Rev Mex Med Transfus 2017, 10, (Supl. 1), S5-S6.

Portillo García, M. L.; López Lom, D.; Cuevas Aguilar, C. J.; Lerma Arias, C. Y.; Grijalva Saavedra, G., Prevalencia de VIH en donadores de la Secretaría de Salud del estado de Chihuahua. Rev Mex Med Transfus 2018, 2018, (11), Supl. 1.

Aguirre-Orozco, A.; Estrada-Mendoza, M. G.; Rodriguez-González, G. A.; Corona-Alfaro, R. A.; Vargas-Carretero, C., Prevalencia de seropositividad en donadores aptos de componentes sanguíneos en el Banco de Sangre del nuevo Hospital Civil de Guadalajara “Dr Juan I Menchaca” en el período comprendido 2015-2018. Rev Mex Med Transfus 2019, 12, (Supl. 1), S7-S8.

González Santos, M. A.; Solano Ricardi, M. M.; Saldaña Vázquez, R., Importancia de la prueba NAT en la seguridad transfusional de los donadores de Banco de Sangre en la UMAE Hospital de Cardiología No. 34. Rev Mex Med Transfus 2019, 12, (Supl. 1), S5.

Guerrero-Garcia, J. J.; Zuniga-Magana, A. G.; Barrera-De Leon, J. C.; Magana-Duarte, R.; Ortuno-Sahagun, D., Retrospective Study of the Seroprevalence of HIV, HCV, and HBV in Blood Donors at a Blood Bank of Western Mexico. Pathogens 2021, 10, (7).
